# Clinical and Laboratory Features of Acute Porphyria: A Study of 36 Subjects in a Chinese Tertiary Referral Center

**DOI:** 10.1155/2016/3927635

**Published:** 2016-11-29

**Authors:** Jing Yang, Qianlong Chen, Hang Yang, Baolai Hua, Tienan Zhu, Yongqiang Zhao, Huadong Zhu, Xuezhong Yu, Li Zhang, Zhou Zhou

**Affiliations:** ^1^Emergency Department, Peking Union Medical College Hospital, Chinese Academy of Medical Sciences and Peking Union Medical College, Beijing, China; ^2^State Key Laboratory of Cardiovascular Disease, Beijing Key Laboratory for Molecular Diagnostics of Cardiovascular Diseases, Diagnostic Laboratory Service, Fuwai Hospital, National Center for Cardiovascular Diseases, Chinese Academy of Medical Sciences and Peking Union Medical College, Beijing, China; ^3^Department of Hematology, Peking Union Medical College Hospital, Chinese Academy of Medical Sciences and Peking Union Medical College, Beijing, China; ^4^Department of Clinical Laboratory, Peking Union Medical College Hospital, Chinese Academy of Medical Sciences and Peking Union Medical College, Beijing, China

## Abstract

Porphyria is a group of eight metabolic disorders characterized by defects in heme biosynthesis. The presentation of porphyria is highly variable, and the symptoms are nonspecific, which accounts in part for delays in establishing a diagnosis. In this study, we report the characteristics of 36 Chinese acute porphyria patients. Most of them were female (33/36), and the median age was 25.3 years (range 18–45 years). The most frequent presenting symptom was abdominal pain (32/36). Hyponatremia was the most common electrolyte abnormality (29/36), and the serum sodium concentration was significantly negatively correlated with convulsion (*p* = 0.00). Genetic testing provided a precise diagnosis of the patients. Genetic analysis of the porphobilinogen deaminase (*PBGD*) gene was performed for 10 subjects. Of them, 9 were found to harbor a mutation in the* PBGD* gene, proving a diagnosis of acute intermittent porphyria, and, in 1 case, a novel Cys209Term mutation was found.

## 1. Introduction

Porphyria is a group of eight metabolic disorders, mainly inherited errors of metabolism characterized by defects in heme biosynthesis. Porphyria is classified into two major categories: (1) acute or inducible porphyria and (2) chronic cutaneous porphyria [1]. The presentation of porphyrias is highly variable, and the symptoms are nonspecific, which accounts in part for delays in establishing a diagnosis. There are four classes of acute hepatic porphyria: acute intermittent porphyria (AIP), hereditary coproporphyria (HCP), variegate porphyria (VP), and porphyria due to severe deficiency of delta-aminolevulinic acid (ALA) dehydratase porphyria (ALADP) [1].

Symptoms of porphyria, which occur in intermittent attacks and may be life-threatening, are caused by the excessive production of porphyrin precursors in the visceral, peripheral, autonomic, and central nervous systems [2]. Genetic testing could provide an early and accurate diagnosis and offer the opportunity to advise affected individuals to avoid drugs and other compounds that may provoke a life-threatening crisis. It could also help to screen asymptomatic family members of patients with acute porphyria. The natural history and clinical and biochemical and genetic features of acute porphyrias have been described in Western countries [3, 4], but, to our knowledge, it has been described in the Chinese population only in case reports [5–12]. In this retrospective study, we analyzed the characteristics of 36 cases of acute porphyria from the Peking Union Medical College Hospital.

## 2. Methods

### 2.1. Study Design and Patients

The study design was approved by the Ethics Committee of the Institutional Review Board at the Peking Union Medical College Hospital (PUMCH).

From January 2013 to May 2016, a total of 36 patients were diagnosed with acute porphyria in the emergency center of the PUMCH. The following diagnostic criteria were used: (1) acute attack symptoms and (2) being positive for urine porphobilinogen (PBG). The clinical features and laboratory evaluation of these 36 patients were retrospectively reviewed.

Among the 36 patients, 10 agreed to have genetic testing for porphobilinogen deaminase (*PBGD*) gene mutations. These 10 patients signed a written consent form stating their acceptance of the genetic testing.

### 2.2. Qualitative Screening Tests of Urinary PBG (Watson-Schwartz Method)

Urine porphobilinogen (PBG) was quantitatively screened using the Watson-Schwartz method. Briefly, urine was added to Ehrlich's reagent (0.7 g dimethylamine borane dissolved in 150 mL concentrated hydrochloric acid and 100 mL water). After 1-2 min, a saturated sodium acetate solution was added, and the nonspecific colors were removed by extraction with chloroform and n-butanol. A positive PBG result was indicated by a distinct pink color in the lower layer [13].

### 2.3. Genetic Testing

The molecular genetic testing of the* PBGD* gene was performed by direct sequencing. All 14 exons of the* PBGD* gene and a minimum of 20 base pairs of flanking intronic DNA for each exon were amplified by polymerase chain reaction (PCR) (Tiangen Biotech, Beijing, China) and subsequently sequenced using the BigDye Terminator Cycle Sequencing Kit version 3.1 (ABI Biosystems) on ABI PRISM 3730 Sequence Analyzer according to the manufacturer's instructions.

### 2.4. Statistics

All statistical analyses were performed using SPSS software (IBM SPSS for Windows, version 16.0, SPSS, Inc., Chicago, IL) for descriptive analysis. Chi-squared tests were employed for categorical variables while parametric Student's *t*-tests or Mann–Whitney *U* tests were used for continuous counterparts, as appropriate. For all comparisons, a *p*-value < 0.05 was considered to represent a significant difference. All statistical tests were 2-sided.

## 3. Results

In this cohort, the gender distribution was predominantly female (33 females, 3 males), and the median age was 25.3 years (range 18–45 years). As for the triggering factors, 28 out of the 36 patients (77.8%) had cyclical attacks linked to the respective patient's menstrual cycle, 2 of the 36 patients (5.6%) had a history of alcohol consumption, 3 of the 36 patients (8.3%) had a history of severe dieting, and 1 patient (2.8%) had taken valproate prior to experiencing acute attacks.

### 3.1. Clinical Features and Laboratory Findings of Acute Porphyria

The clinical manifestations and laboratory findings of acute porphyria are summarized in descending order of frequency in Table 1. Other manifestations may include muscle weakness (*n* = 1), difficulty swallowing (*n* = 1), or sun sensitivity (*n* = 1). A total of 2/36 patients progressed rapidly to acute respiratory insufficiency, and the correct diagnoses were missed for the other patients who were treated for seizures for more than 3 weeks in local hospitals.

Hyponatremia was the most common laboratory abnormality that occurred during the acute attacks (29/36 patients). Other abnormalities observed included transaminase elevation (21/36) and anemia (11/36). The serum sodium concentration was significantly negatively correlated with convulsions (*p* = 0.000, *p* value < 0.05). A total of 14 of 36 patients had severe hyponatremia (<125 mEq/L); all were suspected to have syndrome of inappropriate antidiuretic hormone secretion (SIADH).

Urinary PBG, uroporphyrin, and free erythrocyte protoporphyrin (FEP) were all screened in all patients with acute attacks, the results of which suggested a diagnosis of porphyria. The test results are listed in Table 2.

Genetic analysis for mutations in the* PBGD* gene was performed in 10 patients from 8 families. Of them, 9 patients from 7 families were identified as carriers of known pathogenic mutations, including 2 nonsense and 5 missense mutations (Table 3). Four patients from 4 families had the same mutation (Arg173Trp). Two had been previously reported [5, 11]. Of the mutations identified, Cys209Term is a novel PBGD mutation.

### 3.2. Treatment and Outcome

Once acute porphyria was diagnosed, therapeutic measures were taken for each of the patients, including a high-carbohydrate diet (250–300 g of glucose per day), fluid restriction (<2000 mL per day), and prohibition of drugs that may be harmful to the patient. After acute porphyria was diagnosed, 34 of the 36 patients experienced relief of their symptoms of acute attack, and their lab findings returned to normal values after 6.94 ± 2.16 days (3–14 days, Table 3). One patient, who experienced numbness of the extremities and respiratory paralysis, required 3 months to recover, while another patient (patient 7, Table 3) died as a result of the month-long delay of diagnosis in the local hospital, which resulted in progression to respiratory failure and death within a few days.

## 4. Discussion

Acute porphyria is a group of four gevetic disorders characterized by defective heme biosynthesis. There are four types of acute porphyria: (1) acute intermittent porphyria (AIP, OMIM 176000), which is an autosomal dominant disorder caused by partial deficiency of* PBGD*, the third enzyme in the heme biosynthetic pathway; (2) hereditary coproporphyria (HCP, OMIM 121300), which is caused by a deficiency of coproporphyrinogen oxidase (CPOX), the sixth enzyme in the heme biosynthetic pathway; (3) variegate porphyria (VP, OMIM 176200), which is caused by a deficiency of protoporphyrinogen oxidase (PPOX), the seventh enzyme in the heme biosynthetic pathway; and (4) ALA-dehydratase-deficient porphyria (ADP, OMIM 125270), which is caused by a deficiency of ALA dehydratase, the second enzyme in the porphobilinogen synthase pathway [2].

The clinical manifestations of acute porphyria are diverse and occur as intermittent attacks that may be life-threatening, and they are caused by the excessive production of porphyrin precursors in the visceral, peripheral, autonomic, and central nervous systems [2, 4, 14–16].

In this study, gastrointestinal symptoms were the most frequently reported symptoms (abdominal pain and constipation), with most of the patients diagnosed as having an intestinal obstruction in their first and subsequent attacks. Neurological manifestations were also common (confusion, convulsions, paresis, numbness of the extremities, and respiratory paralysis), and most of the patients were misdiagnosed with epilepsy, but the convulsions may have been related to hyponatremia. Hyponatremia is the most common biochemical manifestation; 14 of 36 patients had severe hyponatremia (<125 mEq/L). Their urine osmolalities were all highly elevated (>100 mosmol/kg), and they were all suspected to have syndrome of inappropriate antidiuretic hormone secretion (SIADH). However, in the absence of serum ADH testing, we could not distinguish SIADH from reduced secretion of antidiuretic hormone [17]. To our knowledge, our report is the largest cohort of subjects with well-documented and well-characterized acute porphyria in China.

The prompt and accurate diagnosis of acute porphyria in China is limited by poor awareness among physicians. Measurements of urinary PBG, ALA, and porphyrins, plasma and fecal porphyrins, and erythrocyte PBG deaminase are important for the diagnosis and typing of porphyria, but, in China, almost none of the hospitals carry out these tests, so additional efforts are required to improve the clinical diagnosis and treatment of porphyria.

Genetic testing may improve the diagnosis and typing of porphyria and may also assist in identifying other carriers of mutations within the patient's family. Of the 36 patients with suspected AIP, 10 submitted to genetic analysis of the* PBGD* gene, and the diagnosis of AIP was subsequently confirmed in 9 of those patients. No mutation was found in the remaining patient (patient 3); however, analysis of the* CPOX* gene (causative gene for HCP) and the* PPOX* gene (causative gene for VP) was not performed. Patient 10, who had a novel mutation in the* PBGD* gene (Cys209Term), presented with common clinical manifestation of porphyria (cyclical attacks of constipation and abdominal pain) but also experienced continuous hypertension, renal dysfunction (creatine: 130–321 *μ*mol/L), and unexplained thrombocytopenia (32–80 × 10^9^ platelets/L), which were aggravated during both of her two pregnancies. Bonkovsky et al. [4] reported no significant associations among clinical or laboratory abnormalities and the general types of porphyria mutations. More data are needed to analyze the relationship between clinical presentation and patient genotype.

In China, hematin is not available to treat acute porphyria. Fortunately, with high carbohydrate intake and the avoidance of harmful drugs, our patients recovered almost fully from their acute attacks, although recovery required weeks or months.

## 5. Conclusion

Patients with acute porphyria attacks are usually women aged 18–45 years who present with severe, recurrent abdominal pain lasting for days. Constipation and hyponatremia are common during these acute attacks. The diagnosis of acute porphyria should be suspected, especially in women who present symptoms linked to their menstrual cycles more than once in the ED. Once suspected, the diagnosis of porphyria can be rapidly established by measuring urinary PBG. In China, genetic testing provides precise diagnosis and typing for the patient. Acute porphyria should be treated with intravenous glucose to avoid considerable morbidity and mortality. The prognosis is good if the disease is recognized early.

## Figures and Tables

**Table 1:** Clinical features and laboratory findings of acute porphyria.

Clinical manifestation (*n* = 36)	

Abdominal pain	32
Cyclical attacks	28
Constipation	26
Confusion	16
Convulsions	12
Tachycardia	10
Paresis	5
Numbness of extremities	3
Hypertension	3
Respiratory paralysis	2

Laboratory findings	

Hyponatremia	29
Severe hyponatremia (<125 mEq/L)	14
Serum Na (mEq/L)	123.0 ± 10.7
Transaminase elevation	22
ALT (U/L)	69.5 ± 56.3
Anemia	10
Hemoglobin (g/L)	117.6 ± 17.9

**Table 2:** Diagnosis of acute porphyria.

Free erythrocyte protoporphyrin (*μ*g/L)	7.5 ± 2.7
PBG positive	36/36
Uroporphyrin positive	22/36
Genetic testing	10

**Table 3:** Summary of genetic analysis of mutations in the *PBGD* gene in 10 patients.

Patient		Clinical features	Total length of hospital stay (days)	Serum Na (mEq/L)	Plasma osmotic pressure	Urine osmotic pressure	Mutation in *PBGD* gene
1	F21	Cyclical attacks, abdominal pain, constipation, confusion, convulsion, tachycardia	13	107	245	447	Ala330Pro [11]
2	F31	Cyclical attacks, abdominal pain, constipation	5	130	280	270	Ala330Pro [11]
3	F24	Cyclical attacks, abdominal pain	3	108	291	585	NO
4	M45	Cyclical attacks, abdominal pain, urine retention	10	109	312	355	Arg173Trp [12]
5	F26	Cyclical attacks, abdominal pain, constipation	9	118	280	732	Arg173Trp
6	F28	Cyclical attacks, abdominal pain, confusion, convulsion	7	112	281	476	Arg173Trp
7	F22	Cyclical attacks, abdominal pain, constipation, respiratory paralysis	11	130			Trp283Term
8	F23	Cyclical attacks, abdominal pain, constipation, confusion, convulsion, tachycardia	10	113	244	730	Trp283Term
9	F34	Cyclical attacks, abdominal pain, sun sensitivity, confusion, convulsion, tachycardia	7	112	241	325	Arg173Trp
10	F33	Cyclical attacks, abdominal pain, constipation, hypertension and renal dysfunction	8	128	314	309	Cys625Term

Patients 1 and 2 were cousins; patients 7 and 8 were sisters.
